# Unlocking Musculoskeletal Anatomy: Enhancing Second-Year Medical Students’ Knowledge Recall and Self-Efficacy with a Physician-Led Ultrasound Session

**DOI:** 10.1007/s40670-025-02414-8

**Published:** 2025-05-20

**Authors:** Nathan Cowan, Abdus Sattar, Qian Wu, Allison N. Schroeder

**Affiliations:** 1 Case Western Reserve University School of Medicine, Cleveland, OH USA; 2 Department of Population and Quantitative Health Sciences, Case Western Reserve University, Cleveland, OH USA; 3 Department of Physical Medicine & Rehabilitation, MetroHealth Systems, Case Western Reserve University, 2500 MetroHealth Drive, Cleveland, OH 44109 USA

**Keywords:** Medical education, Musculoskeletal anatomy, Musculoskeletal ultrasound, Physical examination

## Abstract

**Introduction:**

The purpose of this study was to determine whether exposure to musculoskeletal (MSK) ultrasound (US) enhanced medical students’ knowledge and self-efficacy in understanding basic US principles, MSK anatomy and physical examination, and use of US to evaluate MSK structures.

**Materials and Methods:**

Forty-three second-year medical students were divided into control (*n* = 22) and experimental (*n* = 21) groups. All participants completed a pre-session self-efficacy questionnaire and were encouraged to view a recorded 20-min MSK US lecture. All participants then attended a physician-led 35-min MSK US hands-on scanning session covering the shoulder and knee. The control group took a multiple-choice assessment before the session, while the experimental group took the same assessment after the session. All participants completed a post-session self-efficacy questionnaire. After the session, the physician instructors attended a 30-min Zoom focus group.

**Results:**

Participants’ self-efficacy in understanding basic US principles, knee and shoulder anatomy, physical examination, and sonoanatomy significantly improved after the MSK US session (all *p* < 0.01). Participants who took the content assessment after the MSK US session outperformed participants who took the assessment before the MSK US session (*p* = 0.02). Qualitative open-ended question responses from students and instructor focus group discussion detailed strengths and shortcomings and proposed actionable improvements for future iterations of the MSK US curriculum.

**Conclusion:**

When implemented at a point in the preclinical curriculum where US experience was previously absent, a single, focused physician-led 35-min US session covering the shoulder and knee is an effective adjunct educational tool for preclinical medical students to reinforce US basics, MSK anatomy and physical examination and improve self-efficacy in MSK US scanning.

**Supplementary Information:**

The online version contains supplementary material available at 10.1007/s40670-025-02414-8.

## Introduction

Physicians of various specialties, including emergency medicine, family medicine, physical medicine and rehabilitation (PM&R) and sports medicine, are increasingly utilizing musculoskeletal (MSK) ultrasound (US) at the point-of-care in clinical practice to aid in timely and accurate diagnosis [1–3]. US can be performed quickly upon the initial evaluation of the patient, can be performed dynamically, and is less expensive and has higher superficial spatial resolution than magnetic resonance imaging [4, 5]. US can be used to diagnose acute lower limb injuries on the sidelines of sporting events, confirm suspected neuromusculoskeletal diagnoses in the clinical setting (e.g., tendinosis, tendon tear, ligament tear, muscle tear, fracture, joint effusion, nerve compression), and improve efficacy and safety of injections [6–14]. One of the main limitations of US is that it is an operator-dependent skill, requiring many hours of practice to acquire and interpret images. MSK US can be particularly challenging given that a foundational understanding requires anatomy knowledge of various body regions and that US images need to be collected in non-anatomic planes [15, 16]. As a result, early, frequent, and repetitive exposure to US basics, MSK anatomy, and MSK sonoanatomy are helpful in developing competency.

As medical technology continues to advance, the integration of innovative teaching methodologies has become imperative to nurture well-rounded physicians capable of applying cutting-edge tools, such as US, in clinical practice. Early and frequent exposure to US, starting in medical school, likely enhances the US skills of residents and practicing physicians [17, 18]. Integrating US into medical education is both feasible and beneficial for teaching anatomy, physical examination, physiology, and procedures [19].The integration of US into undergraduate medical education has been shown to improve medical students’ knowledge of anatomy and physical examination of various organ systems, diagnostic accuracy and procedural competency [20–27]. While studies like Lufler et al. have integrated longitudinal US training into anatomy education, many programs still lack scalable interventions that enhance both anatomical knowledge and practical skills without requiring major curricular restructuring [27]. International consensus recommendations support integrating US across basic and clinical science curricula, emphasizing a competency-based model that prepares students for advanced training and patient care [28]. However, MSK US makes up a small portion of these recommendations and the acquisition of MSK US skills predominantly occur during residency and fellowship [3, 29–32]. Several studies have investigated the implementation of a longitudinal MSK US curriculum or standalone MSK US session in undergraduate medical education and have shown benefits in students’ knowledge of anatomical landmarks, performance of physical exam (particularly of soft tissue structures), and understanding of rheumatologic pathology [33–39]. Previous studies by Walrod et al. have explored the impact of MSK US on students’ ability to locate anatomical structures, demonstrating that while a single brief US session did not significantly improve palpation accuracy, repeated exposure led to better performance [34, 36]. Additionally, their findings indicated no significant difference in exam performance between students exposed to MSK US and those who were not. [34, 36].

This study builds on previous research on US integration into anatomy education while offering a distinct contribution by evaluating a concise, physician-led MSK US session within an anatomy curriculum that already allocates time and resources for US instruction. Unlike longitudinal or resource-intensive approaches, this study assesses the impact of a single, focused 35-min MSK US session on second-year medical students’ knowledge recall and self-efficacy (i.e., the task-specific belief in one’s ability to perform an action or achieve a goal). Notably, this intervention occurred within the preclinical MSK block, one year after students had already completed a comprehensive, 2-week head-to-toe cadaveric dissection course. This timing allowed for reinforcement of anatomical knowledge via spaced repetition by re-engaging with relevant MSK structures through a new instructional modality [40]. Integrating MSK US into the later phase of the preclinical curriculum, where US instruction was otherwise absent, provided a unique opportunity to examine how a brief exposure influences students’ understanding of basic US principles, MSK anatomy and self-efficacy when using US to evaluate structures of the knee and shoulder. This session, reinforced anatomy and physical examination skills through hands-on scanning under expert supervision without requiring major curricular changes, offering a practical and scalable strategy for programs constrained by limited time, faculty availability or longitudinal dissection opportunities. It was hypothesized that this MSK US session would enhance students’ understanding of basic US principles, MSK anatomy and physical examination while increasing their comfort in using US to evaluate MSK structures. This hypothesis was based, in part, on the idea that structured multimodal teaching methods (i.e., traditional didactics and hands-on US scanning) are more effective than a single method teaching approach (i.e., traditional didactics only) [41, 42].

## Materials and Methods

### MSK US Course Implementation

#### Current Preclinical Curriculum

The current preclinical education at Case Western Reserve University School of Medicine follows an organ-system-based curriculum consisting of six sequential blocks (Blocks 1–6) and two longitudinal blocks (Blocks 7 and 8) during the first 2 years. The six sequential blocks cover both normal physiology and pathology for all the organ systems (Table 1). Block 7 covers anatomy, radiology, and histopathology for the associated organ systems. Block 8 is comprised of communications workshops, physical exam/clinical reasoning sessions, procedures workshops, and seminar discussions. Between Blocks 1 and 2, there is a 2-week “Anatomy Bootcamp” during which first-year students participate in an intensive full-body cadaveric dissection, excluding the head, neck, and pelvic regions. Prior to our study, Blocks 2–4 incorporated foundational US teaching into their anatomy education; however, the 13-week MSK block (Block 5) which occurs in the first semester of second year did not. Throughout Blocks 2–4, all first-year medical students participate in 14 mandatory 2-h sessions, each consisting of 35 min of gross anatomy lecture, 35 min of radiology, and 35 min of hands-on US structured similarly to the MSK session in this study. This structure provides students with over 8 h of cumulative US experience. The only difference in the format of these 2-h sessions during Block 5 is that the 35 min of hands-on US is replaced with 35 min of MSK physical examination led by physician instructors. The MSK US session in this study was an addition to that standard curriculum.

**Table 1:** Content covered in sequential blocks of preclinical curriculum

Block 1 (1st Year)	Epidemiology, Biostatistics, Public Health, Health Systems and Bioethics
Block 2 (1st Year)	Endocrinology, Reproduction, Cancer, Genetics, Embryology and Molecular/Cell Biology
Block 3 (1st Year)	Nutrition, Biochemistry and Gastroenterology
Block 4 (1st Year)	Cardiology, Pulmonary, Nephrology, Pharmacology and Cell Physiology
Block 5 (2nd Year)	Immunology, Infectious Disease + Microbiology, Hematology/Oncology, Rheumatology, Orthopedics and Dermatology
Block 6 (2nd Year)	Ophthalmology, Otolaryngology, Neurology + Neuroscience and Psychiatry

#### MSK US Session

In order to fill a gap in formal MSK US exposure during Block 5, a concise and high-yield required MSK US session was developed to provide second-year medical students with expert demonstration and hands-on scanning practice covering the shoulder and the knee. This session occurred concurrently to MSK lectures during the MSK block. Before the US session, a 30-min Zoom training was held for the eight volunteer physician instructors (PM&R residents, sports medicine fellows and sports medicine faculty) to cover expectations, session material (Online Resource 1), and student learning objectives.

Before arriving for the US session, all students were encouraged to preview an optional 18-min faculty-developed asynchronous lecture covering the basics of US image acquisition and optimization, the sonographic appearance of MSK structures and common artifacts encountered in MSK US. Students were divided into groups of 4 and each participated in a 35-min session led by one of eight MSK US physician instructors that covered the shoulder and the knee using cart-based Phillips US machines. The covered structures (Table 2) were selected because of their clinical relevance [43], continuity with what was covered in the students’ formal MSK curriculum and frequent appearance of associated anatomy and pathology on standardized USMLE exams. During the 35-min US session, the instructors first provided an 8-min demonstration covering US basics as well as shoulder and knee anatomy and physical exam maneuvers. The remaining time allowed each student approximately 7 min of hands-on scanning time to locate the relevant structures sonographically and identify them by palpation on a volunteer model (one of the four students in the group).

**Table 2:** Shoulder and knee structures covered in the US session

**Shoulder**	**Knee**
Biceps brachii tendon in the bicipital groove	Quadriceps tendon
Acromioclavicular joint	Patella
Supraspinatus	Patellar tendon
Subacromial bursa	Medial joint line + medial meniscus
Glenohumeral joint	Lateral joint line + lateral meniscus

### Participants

All 184 second year medical students in Block 5 at our institution were invited to participate in this study with 45 electing to enroll. First, third- and fourth-year medical students were excluded. Participants were recruited through a series of seven emails from September to October 2023. Informed consent was obtained from all participants prior to their inclusion in the study. The 43 participants that completed all study components received a $20 Amazon gift card at the time of study completion.

### Study Design

**Fig. 1:**
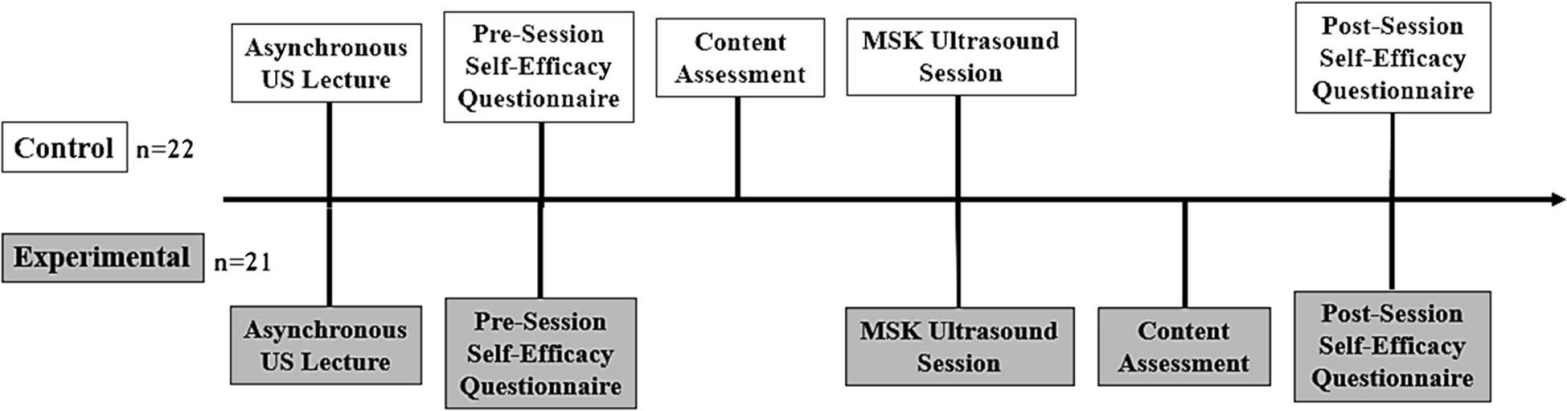
Timeline for the control and experimental groups

Study participants were assigned to either the control or experimental group based on their random assignment of time slots within the current medical school curriculum. Participants in the first three MSK US session time slots were assigned to the control group and those in the last three MSK US session time slots were assigned to the experimental group. All participants in the control and experimental group completed the same pre-session and post-session questionnaires. Prior to the MSK US session, the control group took a faculty-developed content assessment (Online Resource 2) consisting of 25 multiple-choice questions. The experimental group took the same assessment after the MSK US session. Participants were unaware of their content assessment score and did not receive any feedback afterward to avoid unduly influencing their self-efficacy ratings. The study timeline is illustrated in Fig. 1. Study data were securely collected and managed using REDCap electronic data capture tools [44, 45]. The Institutional Review Board at Case Western Reserve University approved this study with exempt status (STUDY20230837).

### Outcome Measures

#### Pre- and Post-Session Questionnaire

All participants took a 15-item pre-session (Online Resource 3) and 23-item post-session (Online Resource 4) self-efficacy questionnaire, developed from surveys in previously published studies examining the effectiveness of US-based instruction in anatomy curricula [27, 35, 46–49]. These questionnaires assessed participants’ comfort with basic US principles, MSK US basics, and MSK anatomy/physical examination. Overall averages for each of the statements were reported using a 5-point Likert Scale where 1 = Strongly Disagree, 2 = Disagree, 3 = Neutral, 4 = Agree and 5 = Strongly Agree. The pre-session questionnaire also asked the participants to indicate whether or not they were involved with the Longitudinal US Elective. This elective spans the first 2 years and provides 20 medical students with additional opportunities to learn how to perform and interpret clinical US examinations (21 h of didactic and hands-on sessions covering various body regions outside of the MSK system). The post-session questionnaire included two open-ended questions gathering feedback about strengths and areas for improvement of the MSK US session.

#### Content Assessment

A 25-question multiple-choice assessment was developed to evaluate participants’ knowledge of basic US principles and shoulder and knee anatomy and physical examination. The questions intended to align with the MSK curricular content and MSK US session content. At the beginning of the content assessment, participants were required to complete an attestation about whether or not they viewed the asynchronous lecture.

#### Instructor Focus Group

The week following the MSK US session, a 30-min Zoom focus group was held with all 8 of the physician instructors to qualitatively discuss strengths and weaknesses of the session. The focus group was moderated by the study’s principal investigator, a Sports Medicine physician (Table 3).

**Table 3:** Instructor focus group questions

1. What were the greatest strengths of the musculoskeletal ultrasound session?
2. How does incorporating ultrasound into the curriculum better reinforce students’ understanding of basic musculoskeletal anatomy and physical exam?
3. Describe the students’ level of participation and engagement
4. Did you notice an improvement in students’ comprehension of the information from the beginning to the end of the session? Why or why not?
5. From a logistical standpoint, what adjustments could make the session run more smoothly?
6. Was there an appropriate amount of time to cover all of the material and allow students to receive adequate hands-on practice? Why or why not?
7. In terms of the content presented, what modifications could create a more educationally beneficial session?
8. Can you comment on the degree of alignment between the depth of material introduced and the expected knowledge of a 2nd-year medical student
9. What ultrasound competencies (scanning or knobology) did students struggle with the most?
10. Would you participate in another iteration of this MSK ultrasound session (Y/N)?

### Statistical Analysis

Within-group comparisons were utilized for the pre- and post-session questionnaire and between-group comparisons were utilized for the content assessment. Pre-and post-session self-efficacy questionnaire data (Online Resources 5 and 6) were summarized as means and 95% confidence intervals. The net differences (ND) of the means for statements 1–15 were calculated by post score minus pre score. The Wilcoxon signed-rank test was applied for comparing these ND at a 5% level of significance (*p* = 0.05). The content assessment raw scores were totaled and converted to a percentage [(*x*/25) × 100] in Excel. The assessments were double-graded to ensure accuracy. The content assessment results were analyzed by comparing the control group with the experimental group using the two-sample t test and Wilcoxon rank-sum test. The Wilcoxon rank-sum test and two-sample t test were applied for comparing mean content assessment scores based on US Elective membership and asynchronous lecture viewership at a 5% level of significance (*p* = 0.05). Data quality and distribution were examined by running frequency analysis and pictorial representations (e.g., histogram, box plot). All statistical analyses were performed using software Stata 18.0. Thematic analysis was used to analyze the two open-ended questions in the post-session self-efficacy questionnaire completed by the students and the focus group discussion transcripts for the instructors. Two researchers independently coded the student and instructor qualitative data to identify common themes, which were then grouped into two categories: “benefits of the MSK session” and “suggestions for further improvement”.

## Results

### Participants

All 184 second-year medical students completed the newly implemented MSK US session (or make-up work). Forty-five students from the class of 184 (24.5%) elected to enroll in the study. Forty-three of those 45 participated in all components of the study: 22 in the control group and 21 in the experimental group (Table 4). Eight students (40%) from the 20-person US elective completed the study. Eight of 8 instructors participated in the post-session Zoom focus group.

**Table 4:** Participant demographics

	**Control (*n* = 22)**	**Experimental (*n* = 21)**
**Ultrasound elective member?**		
Yes	4	4
No	18	17
**Watched pre-recorded lecture?**		
Yes	19	8
No	3	13

### Self-Efficacy Questionnaires

Participants’ self-efficacy in understanding basic US principles, knee and shoulder anatomy and physical examination, and sonoanatomy significantly improved after the MSK US session (Table 5, all *p* < 0.05). Most students thought that US would play a role in their future anatomy education (Pre-Session $\bar{x}$ = 4.23 [4.02,4.44], Post-Session $\bar{x}$ = 4.44 [4.26,4.62], ND = 0.21 [0.00,0.42]) and be a useful skill for medical school graduates regardless of future specialty **(**Pre-Session $\bar{x}$ = 4.35 [4.08,4.62], Post-Session $\bar{x}$ = 4.40 [4.19,4.60]**,** ND = 0.05 [− 0.19,0.28]), but there was not a significant difference before or after the session (*p* = 0.06 and *p* = 0.90, respectively).

**Table 5:** Pre- and post-MSK US session self-efficacy for all participants

**Item**	**Pre-Session Questionnaire**	**Post-Session Questionnaire**	**Net Difference**	***p*-value**
1. Differentiate normal tissues and anatomic landmarks of the knee based on palpation/visual inspection	2.98 (2.63, 3.32)	3.86 (3.63, 4.09)	0.88 (0.57, 1.19)	< 0.01*
2. Differentiate various normal tissues and anatomic landmarks of the shoulder based on palpation/visual inspection	3.09 (2.78, 3.41)	3.86 (3.61, 4.11)	0.77 (0.45, 1.09)	< 0.01*
3. Differentiate various normal tissues and anatomic landmarks of the knee using point of care US	1.93 (1.64, 2.22)	3.67 (3.41, 3.94)	1.74 (1.39, 2.10)	< 0.01*
4. Differentiate various normal tissues and anatomic landmarks of the shoulder using point of care US	1.98 (1.71, 2.24)	3.65 (3.39, 3.91)	1.67 (1.36, 1.99)	< 0.01*
5. Understand basic physics underlying US	3.53 (3.26, 3.81)	3.86 (3.66, 4.06)	0.33 (0.05, 0.60)	0.02*
6. Recognize artifacts on US images relevant to MSK	2.42 (2.08, 2.76)	3.47 (3.17, 3.76)	1.05 (0.72, 1.38)	< 0.01*
7. Handle the US transducer and obtain US images of the knee (utilizing depth, gain, focus)	2.60 (2.25, 2.95)	3.84 (3.56, 4.11)	1.23 (0.91, 1.55)	< 0.01*
8. Handle the US transducer and obtain US images of the shoulder (utilizing depth, gain, focus)	2.58 (2.26, 2.90)	3.77 (3.49, 4.04)	1.19 (0.86, 1.52)	< 0.01*
9. Understand the basic anatomy of the knee	3.37 (3.04, 3.71)	4.02 (3.78, 4.27)	0.65 (0.39, 0.91)	< 0.01*
10. Understand the basic anatomy of the shoulder	3.74 (3.45, 4.04)	4.09 (3.88, 4.30)	0.35 (0.10, 0.60)	< 0.01*
11. Perform physical exam maneuvers for the knee	2.77 (2.47, 3.07)	3.30 (3.01, 3.59)	0.53 (0.23, 0.84)	< 0.01*
12. Perform physical exam maneuvers for the shoulder	2.84 (2.53, 3.14)	3.56 (3.28, 3.84)	0.72 (0.36, 1.08)	< 0.01*
13. Utilize US for supplementing and confirming positive physical exam findings	2.23 (1.93, 2.54)	3.49 (3.17, 3.81)	1.26 (0.93, 1.59)	< 0.01*

Results are reported as the mean (95% confidence interval).

*Indicates statistical significance at a *p* value of < 0.05

On the post-session self-efficacy questionnaire, participants reported that the amount of instruction provided before and during the US workshop was adequate ($\bar{x}$ = 3.91 [3.62,4.19]) and agreed that the session content was relevant to their learning and added value to the MSK anatomy curriculum ($\bar{x}$ = 4.40 [4.23,4.56]) (Online Resource 6).

There were no statistically significant differences in any of the pre- and post-session self-efficacy questionnaire items when stratifying for US Elective membership and asynchronous lecture viewership (Online Resources 5 and 6).

### Content Assessment

Participants in the experimental group had a significantly higher mean score (59.24% [54.43, 64.04]) than those of the control group (50% [44.04, 55.96]) on the content assessment (*p* = 0.02) (Table 6).

**Table 6:** Content assessment scores for all participants with stratification by ultrasound elective membership and lecture viewership

	**Control (*n* = 22)**	**Experimental (*n* = 21)**	***p*-value**
**Overall**	50 (44.04, 55.96)	59.24 (54.43, 64.04)	0.02*
**US Elective Members**	51 (41.45, 60.55)	66 (49.98, 82.02)	0.04*
**US Elective Non-Members**	49.78 (42.45, 57.11)	57.65 (52.35, 62.94)	0.08
**Lecture Viewers**	49.26 (42.46, 56.07)	57.5 (45.52, 69.48)	0.18
**Lecture Non-Viewers**	54.67 (33.98, 75.35)	60.31 (55.53, 65.09)	0.24

Results are reported as the mean (95% confidence interval)

*Indicates statistical significance at a *p*-value of < 0.05

#### Stratified by US Elective Membership

**Fig. 2:**
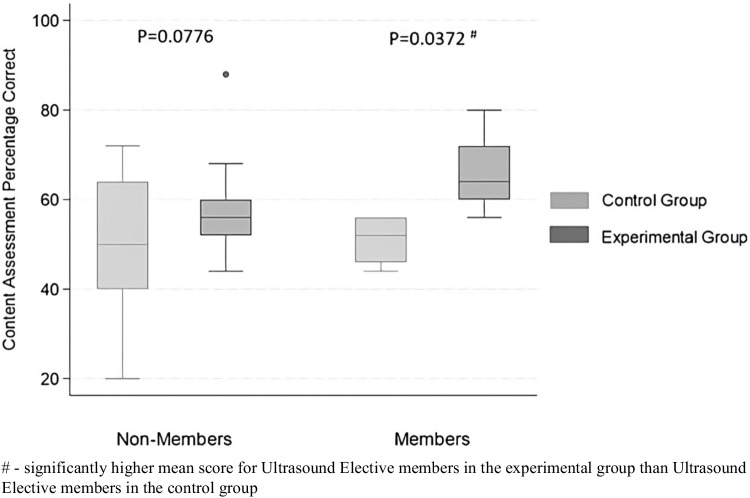
Content Assessment score distributions for control and experimental groups stratified by ultrasound elective membership

The mean content assessment score of US Elective members (58.5% [49.22, 67.78]) was higher than non-members (53.6% [49.06, 58.14]) but this result was not statistically significant (*p* = 0.39) (Online Resource 7). Of participants in the US Elective, those in the experimental group had a significantly higher mean score (66% [49.98, 82.02]) on the content assessment than those in the control group (51% [41.45, 60.55]) (*p* = 0.04) (Table 6, Fig. 2). Of those not in the US Elective, the mean content assessment score was higher in the experimental group (57.65% [52.35, 62.94]) than the control group (49.78% [42.24, 57.11]) but it was not statistically significant (*p* = 0.08) (Table 6, Fig. 2).

#### Stratified by Asynchronous Lecture Viewership

When combining the control and experimental groups, the mean content assessment score of lecture viewers (51.7% [46.00, 57.41]) was lower than those who did not view the lecture (59.25% [54.97, 63.5]), but this result was not statistically significant (*p* = 0.06) (Online Resource 7). Of participants who watched the lecture, there was no significant difference between the mean score of those in the experimental group (57.5% [45.52, 69.48]) versus those in the control group (49.26% [42.46, 56.07]) (*p* = 0.18) (Table 6). There was also no significant difference between the mean score of lecture non-viewers in the experimental group (60.31% [55.53, 65.09]) versus the control group (54.67% [33.98, 75.35]) (*p* = 0.24) (Table 6).

### Qualitative Session Feedback

Analysis of student (Table 7) and instructor (Table 8) qualitative feedback from the open-ended questions and focus group detailed strengths and shortcomings and proposed actionable improvements for future iterations of the MSK US curriculum.

**Table 7:** Summary of student responses from open-ended questions on post-session self-efficacy questionnaire (*n* = 43)

Benefits of the MSK session • The small group size and 4:1 instruction with an expert physician allowed students to have their questions answered & receive assistance performing physical exam maneuvers • The organization of the session and focus on a few, set learning objectives made it possible to comprehend the depth of information covered in the allotted time • The workshop opened their eyes to how point of care US can be used in a sports medicine context • Combining visual inspection and palpation of normal anatomy with utilization of the transducer helped to better cement their understanding • Completing the content assessment before the US workshop may have helped to prime their scanning experience Representative quotes • “*Great resident teaching, interactive session where we got to learn on our own normal anatomy and pathologies which helps retention*.” • “*The preceptors were very knowledgeable about MSK ultrasound and were able to explain concepts in simple terms. Another strength of this session was the depth of information covered in a short amount of time; the session was very efficient*.” • “*Close observation from a faculty member with them having a set curriculum to go through. Was very organized.*”
Suggestions for future improvement • Develop more comprehensive preparatory materials for the students that covered orientation to US images and forecasted the session-specific anatomy given the limited scanning time • Build in more time for in-person practice using gain, depth and focus to optimize images • Split up the session into multiple rooms with appropriate exam tables so models being scanned could assume multiple positions and to have more space and less background noise • Implement additional workshops exploring other joints/anatomical regions and dive more into pathology with reference images • Ensure more standardization of instruction techniques across groups Representative quotes • “*Moving forward, more time would be helpful, as it felt a little rushed, and more time scanning could have been useful. Also, a short review on the different terms used in ultrasound, like gain, depth, hypo/hyperechoic.*” • “*I thought it was a great session, but overall, I don’t think one 30-min session makes me feel confident in this skill. I absolutely found it helpful, but I don’t think I am ‘confident’ in any MSK ultrasound at this point.*” • “*This session could be covered in a much longer duration so that students are able to ask more questions and learn more in depth. I think more time should be spent on each joint. Also, this session should take place in rooms with appropriate chairs so that the patient can easily be in the prone or supine position.*”

**Table 8:** Summary of instructor responses from post-session focus group (*n* = 8)

Benefits of the MSK session • The small group size (4:1 instructor to student ratio) made for a high level of student participation and engagement regardless of interest in entering a MSK-specific specialty • Students’ previous exposure to US in the preclinical curriculum allowed for the session to be focused on MSK US rather than US basics • Students’ ability to recognize different types of tissues improved over the course of the workshop • The session reinforced the basic anatomy being learned in didactics with a focus on functional applications, e.g., dynamic testing of the subacromial space and rotator cuff • Covered an appropriate amount of material given the length of the session at an appropriate level of depth for a second-year medical student Representative quotes • “*I think it was great to have small groups so they could get their hands on the ultrasound and see things up close. It made it feel more personalized and manageable.*” • “*They were able to quickly identify, ‘Oh, that’s muscle. That’s tendon.’ And when we got to a new area, they could apply what they learned earlier. It made the concepts stick more.*” • “*It just reinforces, you know, what they’re learning in their didactics with actually more functional applications.*”
Suggestions for future improvement • Ensure students wear clothing that will not restrict their ability to have their joints scanned and recruit dedicated models rather than rotating students through the role of standardized patient • Include slightly more time per station to avoid rushing students and allow for scanning additional high-yield anatomic areas such as the carpal tunnel and medial/lateral epicondyle of the elbow • Create a short video tutorial (in addition to the other preparatory materials) demonstrating the structures that would be scanned to streamline the US workshop • Require the instructors to show up 10–15 min before the session to orient themselves to the US machines and transducers Representative quotes • “*I think the amount of time was appropriate, but we could have maybe had five more minutes for each joint.*” • “*Maybe doing a quick tutorial video of actually scanning the structures that we’re going to demonstrate… a quick, like, ten-minute video beforehand so they at least have some baseline might be helpful.*” • “*If we were there, like, ten minutes earlier, just say a walk through using that machine. Because I feel like a lot of time was wasted in the first groups, flipping through the different views.*”

## Discussion

Despite MSK conditions (e.g., arthritis, tendinopathy) being among the most prevalent health issues, MSK instruction is often limited in undergraduate medical curricula, with MSK US rarely included [29, 50, 51]. Compressed preclinical timeframes, limited faculty expertise and availability, and the high cost of US equipment are frequently cited as significant barriers to integrating new educational sessions or tools into established undergraduate medical curricula [52–55]. This study explored the implementation of a brief 35-min MSK US session, providing hands-on learning with a technology that is increasingly recognized as both an effective teaching aid and powerful clinical diagnostic tool across various specialties [1–3, 20–26, 33–39]. Students who participated in the session demonstrated significant improvement in their self-efficacy regarding US basics and knee and shoulder anatomy, physical examination, and MSK US sonographic principles Students had improved self-efficacy in understanding US basics and knee and shoulder anatomy, physical examination, and sonoanatomy after completing the session. Students who completed the MSK US session performed significantly higher on the 25-question content assessment than those who did not, underscoring the session’s value as an adjunct to the established curriculum. Overall, in line with our hypothesis, this focused faculty-led MSK US session allowed for a multimodal approach to preclinical medical student MSK education (combining a hands-on US scanning session with traditional didactics) and enhanced students’ understanding of basic US principles, MSK anatomy and physical examination while increasing their comfort in using US to evaluate MSK structures.

This study offers a distinct contribution by evaluating the impact of a single, concise MSK US session delivered during the second year of medical school—approximately one year after students completed a comprehensive cadaveric dissection course. By revisiting previously dissected knee and shoulder structures through a new instructional modality, the session reinforced anatomical knowledge via spaced repetition, a strategy known to enhance long-term retention. Unlike longitudinal or resource-intensive interventions, this session allowed for multimodal learning and was embedded into an existing MSK block without requiring major curricular changes, addressing a gap where formal US instruction was otherwise absent. This timing allowed students to apply foundational US skills in a clinically relevant, hands-on context, enhancing both understanding and self-efficacy in alignment with competency-based education goals.

Analysis of qualitative feedback from students and instructors revealed strengths of the MSK US session, including the small 4:1 student-to-instructor ratio, which allowed for personalized instruction, real-time feedback, and hands-on practice. Students appreciated the session’s organized structure and clear learning objectives, which aided retention within the limited time frame. Many also highlighted the benefit of combining US imaging with physical examination and palpation of normal anatomy. However, students suggested more time for refining scanning techniques (e.g., adjusting gain, depth, and focus) and additional time per station to explore other structures such as the carpal tunnel and epicondyles of the elbow. Instructors echoed the need for slight modifications, such as requiring students to wear appropriate clothing for scanning and recruiting dedicated models to improve scanning consistency. Finally, both groups proposed creating a brief instructional video covering the anatomical regions that would be scanned to enhance familiarity before hands-on practice.

Several other studies have explored the integration of MSK US exposure into first-year undergraduate medical education curricula. One study by De Vries et al. demonstrated a significant improvement in students’ shoulder palpation skills and their ability to palpate the long head of the biceps tendon. [37] A simultaneous study by Walrod et al. showed that MSK US improved students’ ability to locate the long head of the biceps tendon by physical examination, but it did not significantly improve students ability to palpate bony structures including the medial and lateral joint lines or the AC joint. [34] A follow-up study by Walrod et al. showed that a single 15-min US session did not improve students’ ability to palpate soft tissue structures of the upper and lower limbs, however, palpation accuracy improved following repeated exposure with an additional 15-min US session. [36] To address these findings, our session was designed to last 35 min, exceeding the 30-min threshold suggested by prior research. Whereas the studies by Walrod et al. found no significant difference in exam (content and practical) performance between students exposed to MSK US and those who were not, our study demonstrated that even brief MSK US exposure improved immediate recall of knowledge pertaining to US basics, MSK anatomy, physical examination, and sonoanatomy and improved self-efficacy. However, long-term knowledge retention was not evaluated to see if this improvement was sustained.

Repeated exposure to a topic is known to improve medical students’ knowledge retention, and this likely applies to US education as well [56–58]. In our study, students enrolled in the Ultrasound Elective who completed the MSK US scanning session scored significantly higher on the content assessment than their peers in the same elective who had not yet completed the session. Interestingly, among students not enrolled in the Ultrasound Elective, content assessment scores were not significantly different, even after completing the scanning session. This suggests that a short MSK US session may be especially beneficial for students with a stronger baseline understanding and familiarity with general US principles. These students may be able to focus more on reinforcing US basics and acquiring MSK-specific knowledge rather than having to learn US fundamentals during the session. Further research is needed to explore the factors driving this association (e.g., the threshold of baseline US exposure needed to appreciate this benefit) and to better understand the benefits of longitudinal US exposure.

Several limitations should be considered when interpreting the study findings. First, the assessment of knowledge recall was limited to short-term outcomes, without follow-up testing weeks or months later to evaluate long-term retention of US basics, knee and shoulder anatomy, physical examination, and sonoanatomy. This restricts the ability to draw conclusions about the lasting impact of this educational intervention. Second, the relatively small sample size limited the study’s statistical power, and a larger sample may have provided more robust results. Third, the 35-min MSK US session offered only brief exposure, and longer or repeated sessions were not examined to determine whether they would have enhanced knowledge retention and self-efficacy. Fourth, a significant proportion of participants (37.2%) did not watch the pre-recorded lecture with more students in the control group than in the experimental group watching the lecture, which may have impacted students’ baseline knowledge and performance on the content assessment, potentially influencing study outcomes. Requiring preparatory work as an eligibility criterion for study participation could further minimize sources of bias. Fifth, all participating students had prior US exposure during earlier preclinical blocks, potentially influencing their baseline comfort with US equipment and anatomy. However, all students received the same preclinical exposure, and we attempted to adjust for the confounder of those who had additional exposure through completion of the US elective. Nevertheless, prior US exposure may have confounded observed improvements in self-efficacy and knowledge, making it difficult to isolate the impact of the MSK US session alone. Future studies should consider controlling for or stratifying based on prior US exposure. Sixth, the administration of the post-session self-efficacy questionnaire immediately after the content assessment may have introduced bias. Although students did not receive their assessment scores, the proximity of these two outcome measures might have inflated of deflated their self-efficacy ratings based on their perceived performance. Temporally separating the content assessment and post-session self-efficacy questionnaire may mitigate this effect. Last, the 25-question content assessment, while designed for time efficiency, may have lacked the depth or length to detect significant differences between compared groups. These limitations underscore the need for cautious interpretation and point to future research opportunities, including evaluating long-term impacts, optimizing session length, controlling for prior US exposure, adjusting timing of outcome measures and refining assessment methods for MSK US in undergraduate medical education.

There are several future studies that could be performed to assess implementation of an MSK US session (or multiple sessions) into the US curriculum. Future studies could assess knowledge retention by having the students take the same content assessment several months after the MSK US session. Additionally, MSK end-of-block exam scores could be compared between years to determine if the short session had any impact on the students overall scores. Additionally, we could investigate the implementation of additional MSK US sessions or the implementation of longer sessions within the MSK block to determine the optimum stimulus for knowledge enhancement. Another future longitudinal study could replicate this design by assessing baseline knowledge and self-efficacy in US at the beginning of students’ preclinical years followed by a comprehensive evaluation at the end of their 2 years of exposure to US across all body systems. This design could more accurately quantify the cumulative impact of US training during the preclinical curriculum.

## Conclusion

This study highlights the value of integrating a brief, faculty-led MSK US session into the undergraduate medical education MSK curriculum. A 35-min session significantly boosted students’ self-efficacy and knowledge of US basics and MSK anatomy, physical examination, and sonographic principles. Unlike prior studies using longitudinal or resource-intensive approaches, this intervention was embedded into an existing MSK block without requiring major curricular restructuring. By leveraging students’ prior US exposure later in the preclinical curriculum, the study demonstrates that even a single, well-structured session can serve as an effective adjunct to traditional anatomy and physical exam education. These findings support the feasibility and educational value of integrating MSK US into time- and resource-limited academic settings.

## Supplementary Information

Below is the link to the electronic supplementary material.

Supplementary file1 (DOCX 20 KB)

Supplementary file2 (DOCX 2841 KB)

Supplementary file3 (DOCX 15 KB)

Supplementary file4 (DOCX 15 KB)

Supplementary file5 (DOCX 19 KB)

Supplementary file6 (DOCX 22 KB)

Supplementary file7 (DOCX 48 KB)

## Data Availability

Data collected will be available upon reasonable request for a few years.
