# Supplementary material for: Unlocking Musculoskeletal Anatomy: Enhancing Second-Year Medical Students’ Knowledge Recall and Self-Efficacy with a Physician-Led Ultrasound Session
Source: Med Sci Educ. 2025 May 20;35(4):2063–74. doi: 10.1007/s40670-025-02414-8 (PMC12532992; doi:10.1007/s40670-025-02414-8)
Supplement: Supplementary file 1 — Supplementary file1 (DOCX 20 KB) [file 40670_2025_2414_MOESM1_ESM.docx]

Article Title - Unlocking Musculoskeletal Anatomy: Enhancing Second-Year Medical Students’ Knowledge Recall and Self-Efficacy with a Physician-Led Ultrasound Session

Journal Name – Medical Science Educator

Author Names – Nathan Cowan, BS^;^ Abdus Sattar, PhD, LLM; Qian Wu, BMS; Allison N. Schroeder, MD

Corresponding Author E-Mail & Affiliation – [aschroe1@alumni.nd.edu](mailto:aschroe1@alumni.nd.edu) ; Department of Physical Medicine & Rehabilitation, MetroHealth Systems, Case Western Reserve University

**Supplemental Material 1**

*MSK US Session Lesson Plan with Shoulder & Knee Structures*

**Shoulder:**

**Short Demo (5 min), may need to briefly review US basics**

- **Biceps tendon with dynamic maneuver (Student #1)**
- **AC joint (Student #1)**
- **Supraspinatus/subacromial bursa (Student #2)**
- **Glenohumeral joint (Student #2)**

**Knee:**

**Short Demo (3 min)**

- **Quadriceps muscle & tendon (Student #3)**
- **Suprapatellar recess (aka bursa) (Student #3)**
- **Patella and patellar tendon (Student #3)**
- **Medial joint line /meniscus / MCL (Student #4)**
- **Lateral joint line / meniscus (Student #4)**

Learning Points:

OVERALL: Goal is to improve basic MSK US knowledge, reinforce pertinent shoulder and knee anatomy and physical examination pearls

**General:**

- Describe transducer selection while scanning
  - Higher frequency, lower wavelength – superficial imaging (linear)
  - Lower frequency, higher wavelength – deep imaging (curvilinear)
- While scanning, describe optimization of depth/gain(brightness)/focus to improve image
- Review common ultrasound terms – hyperechoic, isoechoic, hypoechoic, anechoic

**Biceps tendon with dynamic maneuver (Student #1)**

- Point out that the biceps tendon is best imaged with palm up (supination) in neutral or slight external rotation
- Review change in positioning of biceps tendon with internal rotation (moves tendon medially) and external rotation (moves tendon laterally) of the shoulder
- Review anisotropy when imaging the biceps tendon – occurs when beam is not perpendicular to the tendon fibers and the structure looks dark (mimics tear)

**AC joint (Student #1)**

- Review scarf test when imaging the AC joint
- Review that the AC joint is formed between the clavicle and acromion (scapula)

**Supraspinatus/subacromial bursa (Student #2)**

- Point out posterior acoustic shadow artifact when imaging the supraspinatus and subacromial/subdeltoid bursa
- Recall that supraspinatus helps to abduct the arm
- Correlate imaging of the supraspinatus and subacromial subdeltoid bursa with Hawkin’s maneuver for impingement - abduction and internal rotation

**Glenohumeral joint (Student #2)**

- Review that the glenohumeral joint is an articulation between the humeral head of the humerus and the glenoid of the scapula

**Quadriceps muscle & tendon (Student #3)**

- Review normal appearance of muscle – hypoechoic with hyperechoic fascial planes

**Suprapatellar recess (aka bursa) (Student #3)**

- Review the location of the suprapatellar recess (deep to quadriceps tendon)

**Patella and patellar tendon (Student #3)**

- Review normal appearance of bone and posterior acoustic shadow
- Review normal appearance of tendon – hyperechoic and fibrillar

**Medial joint line / meniscus / MCL (Student #4)**

- Examine the location of the medial joint line in relation to the patella to aid in identifying it on palpation – with knee flexed to 90 (common way to examine), joint line is near inferior pole of patella
- Remind students of the function of the meniscus - provide cushion to the joint

**Lateral joint line / meniscus (Student #4)**

- Examine the location of the lateral joint line in relation to the patella to aid in identifying it on palpation. – with knee flexed to 90 (common way to examine), joint line is near inferior pole of patella
- When examining the joint lines, not that the femur sits proximally and the tibia distally with the meniscus in between
