# Supplementary material for: Unlocking Musculoskeletal Anatomy: Enhancing Second-Year Medical Students’ Knowledge Recall and Self-Efficacy with a Physician-Led Ultrasound Session
Source: Med Sci Educ. 2025 May 20;35(4):2063–74. doi: 10.1007/s40670-025-02414-8 (PMC12532992; doi:10.1007/s40670-025-02414-8)
Supplement: Supplementary file 2 — Supplementary file2 (DOCX 2841 KB) [file 40670_2025_2414_MOESM2_ESM.docx]

Article Title - Unlocking Musculoskeletal Anatomy: Enhancing Second-Year Medical Students’ Knowledge Recall and Self-Efficacy with a Physician-Led Ultrasound Session

Journal Name – Medical Science Educator

Author Names – Nathan Cowan, BS^;^ Abdus Sattar, PhD, LLM; Qian Wu, BMS; Allison N. Schroeder, MD

Corresponding Author E-Mail & Affiliation – [aschroe1@alumni.nd.edu](mailto:aschroe1@alumni.nd.edu) ; Department of Physical Medicine & Rehabilitation, MetroHealth Systems, Case Western Reserve University

**Supplementary Material 2**

*MSK US Content Assessment & Answer Key*

Attestation: By answering yes, you attest that you have watched the pre-recorded lecture in its entirety prior to attending today’s ultrasound session.

□ Yes

□ No

1. Which of the following is true about the frequency of ultrasound waves used in musculoskeletal imaging? a) Higher frequency ultrasound waves penetrate deeper into tissues b) Lower frequency ultrasound waves provide higher resolution images c) Higher frequency ultrasound waves provide higher resolution images d) Frequency does not affect image resolution e) Higher frequency ultrasound waves also have a lower wavelength
2.
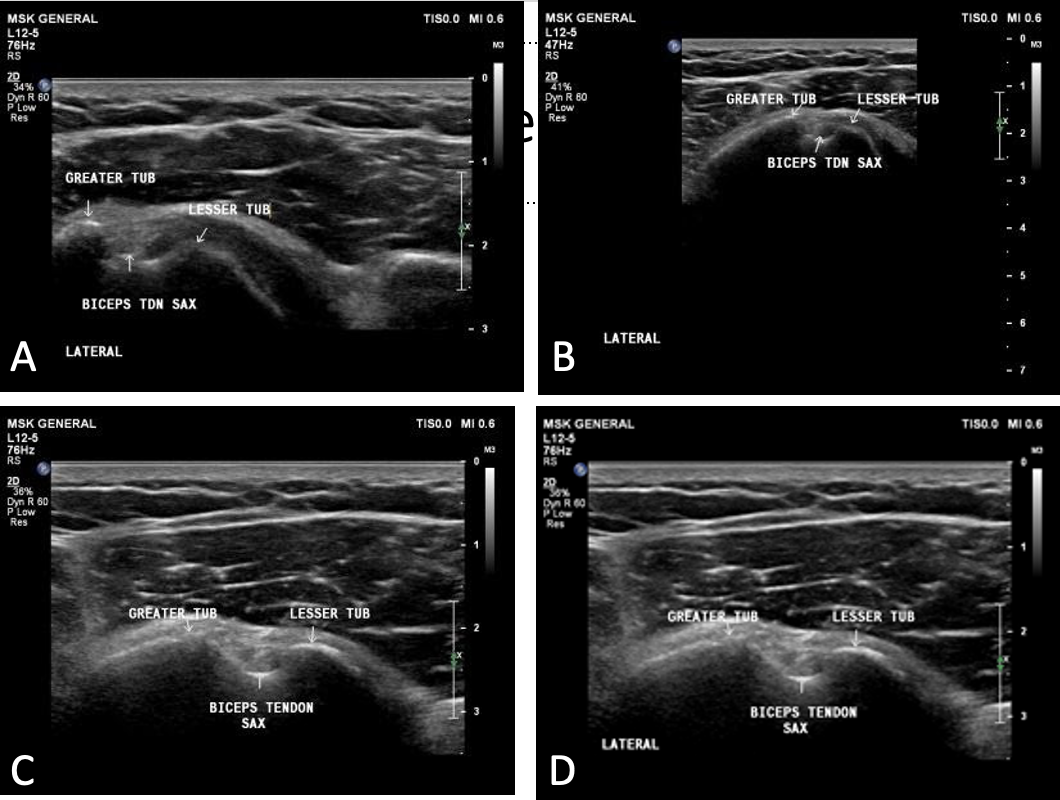
Which image is properly labeled and optimized?

1. In ultrasound imaging, if a structure is hyperechoic, what does it mean? It is a) Brighter (or whiter) compared to surrounding tissues b) Darker or (blacker) compared to surrounding tissue c) Gray in color d) Definitely a tendon e) Transparent
2. What does the "anisotropy phenomenon" refer to in musculoskeletal ultrasound imaging? a) The ability of certain tissues to reflect sound waves. b) The ability of certain tissues to change their shape under pressure. c) The tendency of tendons to appear hypoechoic on ultrasound. d) The tendency of tendons to change their echogenicity with different transducer orientations e) The tendency of a structure to reflect different amounts of sound waves at different depths
3. In ultrasound imaging, the term "gain" refers to: a) The depth at which the image is focused. b) The amplification of received echoes to improve image brightness. c) The size of the ultrasound transducer. d) The frequency of the ultrasound waves emitted. e) The amount of through transmission that occurs
4. What most accurately describes the sonographic appearance of the quadriceps muscles? a) Hypoechoic with hyperechoic fascial planes b) Hyperechoic and homogenous c) 4 distinct muscles that are isoechoic to the quadriceps tendon d) Normal muscles have heavy intramuscular Doppler flow
5. What best describes the sonographic appearance of the patellar tendon imaged in long axis? a) Hyperechoic with fibrillar pattern b) Hypoechoic with linear striations c) Anechoic with fluid-filled spaces d) Isoechoic with homogeneous texture
6. What is the dynamic component of musculoskeletal ultrasound? a) The ability to assess joint mobility b) The ability to detect blood flow in vessels c) The ability to evaluate muscle contractility d) The ability to measure strain in tendons
7. What is the name of the fluid-filled sacs located around the shoulder and knee joints? a) Tendons b) Bursae c) Ligaments d) Cartilage
8.
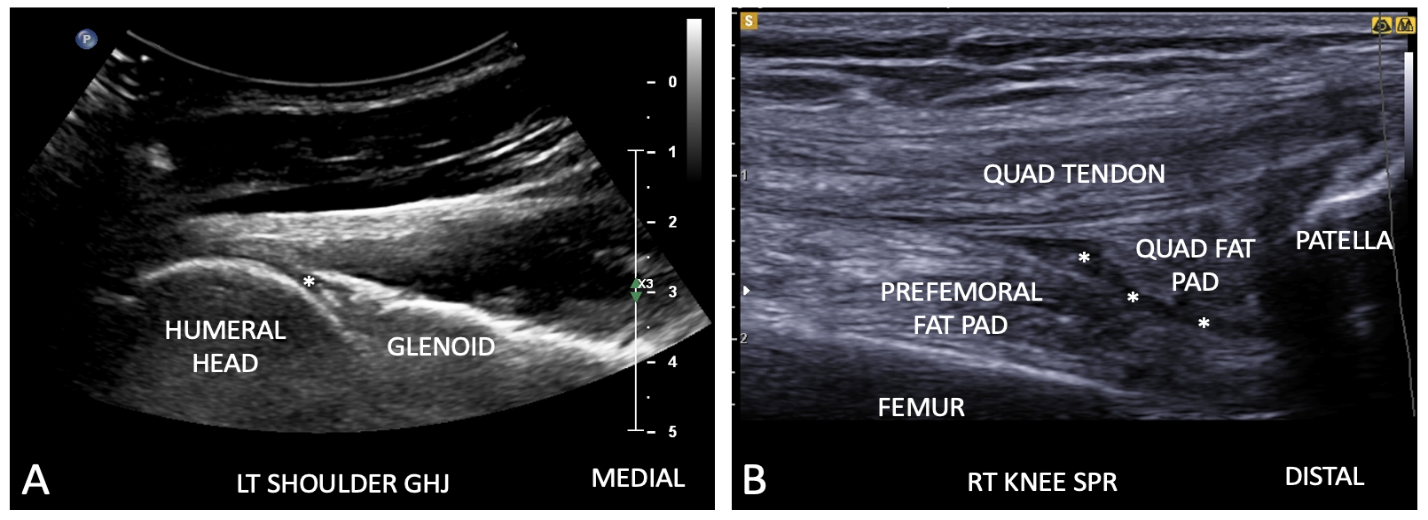
Which statement is accurate regarding these images?

a) The transducer used to obtain image A has a higher frequency than the transducer used in image B b) The transducer used to obtain image A has a lower wavelength than the transducer used in image B c) The transducer used to obtain image A is a linear transducer d) The transducer used in image B in on beam steering mode e) The transducer used to obtain image A has a lower frequency than the transducer used to obtain image B

1. Best visualization of the long head of the biceps tendon occurs with what arm positioning? a) Shoulder in neutral, elbow flexed to 90 degrees and forearm pronated b) Shoulder in external rotation, elbow flexed to 90 degrees and forearm pronated c) Shoulder in external rotation, elbow flexed to 90 degrees and forearm supinated d) Shoulder in neutral, elbow flexed to 90 degrees and forearm supinated e) You can see it well no matter the arm position
2. What is the primary function of the rotator cuff muscle shown in this image (asterisk)?


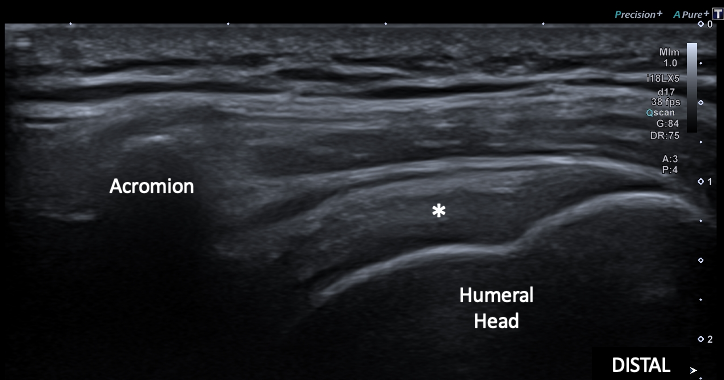


1. Flexion b) Extension c) Internal rotation d) External rotation e) Abduction
2. Which ultrasound artifacts (between the solid arrows in the image below) seen in MSK imaging are accurately identified?


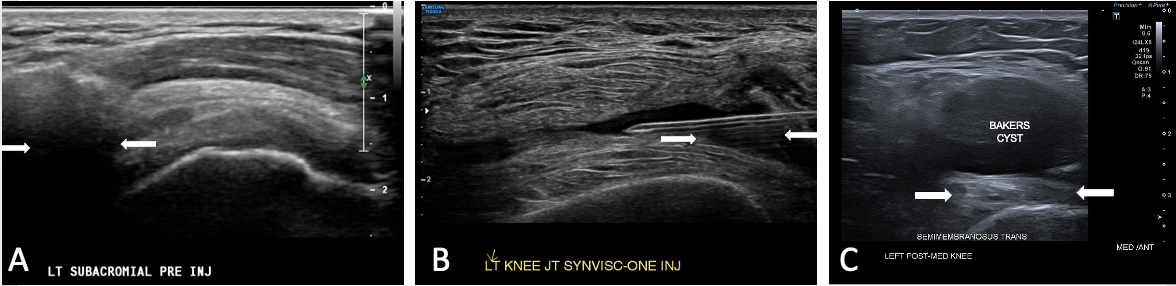


1. A=Increased through transmission, B=Posterior acoustic shadow b) B=Reverberation artifact, C=Posterior acoustic shadow c) B=Posterior acoustic shadow, C=Reverberation artifact d) A=Posterior acoustic shadow, C=Increased through transmission
2. Which structure cushions and provides shock absorption in the knee joint? a) Articular cartilage b) Menisci c) Synovial fluid d) Bursae e) ACL
3. What is the function of the patellar tendon? a) It connects the quadriceps muscle to the tibia b) It connects the hamstrings muscle to the tibia c) It provides stability to the lateral aspect of the knee joint d) It cushions the knee joint during weight-bearing activities e) It allows the knee to flex
4. Which maneuver is used to assess shoulder impingement? a) Obrien’s test b) Apprehension test c) Lachman test d) McMurray test e) Hawkin’s test
5. With the knee flexed to 90 degrees, the medial and lateral joint line are _______ in relation to the patella: a) near the superior pole of the patella b) near the middle of the patella c) near the inferior pole of the patella d) it varies highly between different individuals e) in the same position in relation to the patella if the knee were to be extended
6. The scarf test causes which 2 bones (that make up a joint in the shoulder region) to approximate: a) Clavicle and scapula b) Humerus and scapula c) Scapula and sternum d) Clavicle and sternum e) Humerus and clavicle
7. When visualizing the posterior glenohumeral joint under ultrasound, which shoulder movement will allow for better visualization of a shoulder joint effusion? a) Internal rotation b) External rotation c) Extension d) Flexion e) Abduction
8. The suprapatellar recess communicates with the knee joint and: a) appears anechoic and filled with fluid in a normal knee joint b) sits anterior to the patella c) has fluid within it when an extra-articular knee injury occurs d) is deep to the quadriceps tendon e) is immediately anterior and adjacent to the femur
9. When assessing surface anatomy and performing palpation on physical examination, external rotation of the shoulder will move the long head of the biceps tendon in which direction (compared to shoulder in neutral)? a) Laterally b) Medially c) Superficial d) Deep e) Superior
10. What is the triangular hyperechoic homogeneous structure that sits deep to the MCL and between the femur and tibia? a) Medial plica b) Medial meniscus c) Meniscofemoral ligament c) Meniscotibial ligament e) Semimembranosus tendon
11. Which dynamic maneuver is appropriated paired with its anatomic correlation? a) Shoulder internal and external rotation – infraspinatus tendon and bursa slide under the coracoid process b) Cross body adduction – posterior labrum is pinched between the glenoid and humeral head c) Internal rotation – long head of biceps tendon is pinched under the acromion d) Shoulder abduction and internal rotation – supraspinatus tendon is pinched under the acromion
12. Which bone forms the articulation with the humerus to create the shoulder joint? a) Scapula b) Clavicle c) Acromion d) Radius e) Coracoid
13. Which image is accurately labeled? (POP=popliteus tendon; LM=lateral meniscus)


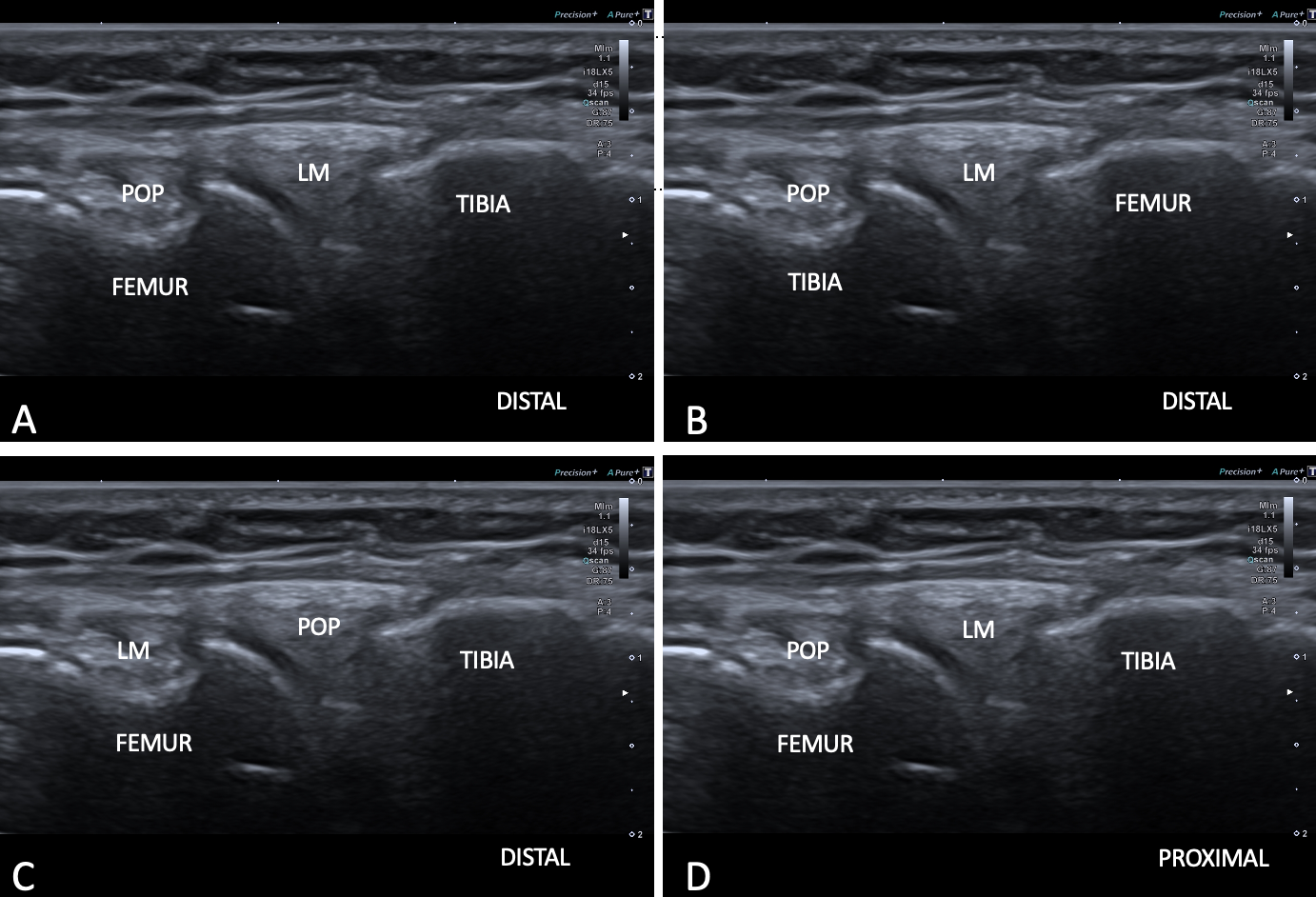


Answer Key:

1. C
2. D
3. A
4. D
5. B
6. A
7. A
8. A
9. B
10. E
11. D
12. E
13. E
14. B
15. A
16. E
17. C
18. A
19. D
20. D
21. A
22. B
23. D
24. A
25. A
