# Supplementary material for: Unlocking Musculoskeletal Anatomy: Enhancing Second-Year Medical Students’ Knowledge Recall and Self-Efficacy with a Physician-Led Ultrasound Session
Source: Med Sci Educ. 2025 May 20;35(4):2063–74. doi: 10.1007/s40670-025-02414-8 (PMC12532992; doi:10.1007/s40670-025-02414-8)
Supplement: Supplementary file 4 — Supplementary file4 (DOCX 15 KB) [file 40670_2025_2414_MOESM4_ESM.docx]

Article Title - Unlocking Musculoskeletal Anatomy: Enhancing Second-Year Medical Students’ Knowledge Recall and Self-Efficacy with a Physician-Led Ultrasound Session

Journal Name – Medical Science Educator

Author Names – Nathan Cowan, BS^;^ Abdus Sattar, PhD, LLM; Qian Wu, BMS; Allison N. Schroeder, MD

Corresponding Author E-Mail & Affiliation – [aschroe1@alumni.nd.edu](mailto:aschroe1@alumni.nd.edu) ; Department of Physical Medicine & Rehabilitation, MetroHealth Systems, Case Western Reserve University

**Supplementary Material 4**

*Post-Session Self-Efficacy Questionnaire*

To what extent do you agree or disagree with the following statements: (Strongly Disagree, Disagree, Neutral, Agree, Strongly Agree)

1. As of today, I feel confident in my ability to differentiate various normal tissues and anatomic landmarks of the knee based on palpation/visual inspection*
2. As of today, I feel confident in my ability to differentiate various normal tissues and anatomic landmarks of the shoulder based on palpation/visual inspection*
3. As of today, I feel confident in my ability to differentiate various normal tissues and anatomic landmarks of the knee using point of care ultrasound*
4. As of today, I feel confident in my ability to differentiate various normal tissues and anatomic landmarks of the shoulder using point of care ultrasound*
5. I understand the basic physics underlying ultrasound as an imaging modality*
6. I am confident in my ability to recognize artifacts on ultrasound images relevant to MSK*
7. I am confident in my ability to handle the ultrasound transducer and obtain ultrasound images of the knee (utilizing depth, gain, focus)*
8. I am confident in my ability to handle the ultrasound transducer and obtain ultrasound images of the shoulder (utilizing depth, gain, focus)*
9. I am confident in my understanding of the basic anatomy of the knee*
10. I am confident in my understanding of the basic anatomy of the shoulder*
11. I am confident in my ability to perform physical exam maneuvers for the knee*
12. I am confident in my ability to perform physical exam maneuvers for the shoulder*
13. I am confident in my ability to utilize ultrasound for supplementing and confirming positive physical exam findings*
14. Ultrasound will play a role in the future of anatomy education*
15. Ultrasound is a useful skill for graduates regardless of future speciality*
16. The ultrasound workshop helped improve my comprehension of the anatomy of the knee*
17. The ultrasound workshop helped improve my comprehension of the anatomy of the shoulder*
18. The ultrasound workshop helped improve my physical examination skills for the knee*
19. The ultrasound workshop helped improve my physical examination skills for the shoulder*
20. The ultrasound workshop improved my ability to orient myself to an sonographic image of the knee*
21. The ultrasound workshop improved my ability to orient myself to an sonographic image of the shoulder*
22. The amount of instruction provided prior to and during the MSK ultrasound session was adequate*
23. This session’s content was relevant to my learning and added value to my Block 5 GARLA experience*

Open-Ended Questions for Post-Session Questionnaire

1. What were the strengths of today’s session?
2. What could be improved upon for next time?

* = Required field
