# Supplementary material for: Unlocking Musculoskeletal Anatomy: Enhancing Second-Year Medical Students’ Knowledge Recall and Self-Efficacy with a Physician-Led Ultrasound Session
Source: Med Sci Educ. 2025 May 20;35(4):2063–74. doi: 10.1007/s40670-025-02414-8 (PMC12532992; doi:10.1007/s40670-025-02414-8)
Supplement: Supplementary file 5 — Supplementary file5 (DOCX 19 KB) [file 40670_2025_2414_MOESM5_ESM.docx]

Article Title - Unlocking Musculoskeletal Anatomy: Enhancing Second-Year Medical Students’ Knowledge Recall and Self-Efficacy with a Physician-Led Ultrasound Session

Journal Name – Medical Science Educator

Author Names – Nathan Cowan, BS^;^ Abdus Sattar, PhD, LLM; Qian Wu, BMS; Allison N. Schroeder, MD

Corresponding Author E-Mail & Affiliation – [aschroe1@alumni.nd.edu](mailto:aschroe1@alumni.nd.edu) ; Department of Physical Medicine & Rehabilitation, MetroHealth Systems, Case Western Reserve University

**Supplemental Material 5**

*Descriptive Statistics of the Pre-Session Self-Efficacy Questionnaire for All Participants with Stratification by Ultrasound Elective Membership and Lecture Viewership*

Results are reported as the Mean (95% Confidence Interval)

| **Item** | **Overall** | **US Elective Members** | **US Elective Non-Members** | **Lecture Viewer** | **Lecture**  **Non-Viewers** |
| --- | --- | --- | --- | --- | --- |
| 1. As of today, I feel confident in my ability to differentiate various normal tissues and anatomic landmarks of the knee based on palpation/visual inspection | 2.98  (2.63, 3.32) | 3.13  (2.18, 4.07) | 2.95  (2.55, 3.33) | 2.93  (2.52, 3.34) | 3.06  (2.38, 3.75) |
| 2. As of today, I feel confident in my ability to differentiate various normal tissues and anatomic landmarks of the shoulder based on palpation/visual inspection | 3.09  (2.78, 3.41) | 3.25  (2.66, 3.84) | 3.06  (2.69, 3.43) | 3.07  (2.66, 3.48) | 3.13  (2.58, 3.67) |
| 3. As of today, I feel confident in my ability to differentiate various normal tissues and anatomic landmarks of the knee using point of care US | 1.93  (1.64, 2.22) | 2.25  (1.38, 3.12) | 1.86  (1.54, 2.17) | 2.07  (1.70, 2.45) | 1.69  (1.22, 2.15) |
| 4. As of today, I feel confident in my ability to differentiate various normal tissues and anatomic landmarks of the shoulder using point of care US | 1.98  (1.71, 2.24) | 2.13  (1.30, 2.95) | 1.94  (1.65, 2.23) | 2.07  (1.73, 2.42) | 1.81  (1.37, 2.26) |
| 5. I understand the basic physics underlying US as an imaging modality | 3.53  (3.26, 3.81) | 3.63  (2.63, 4.62) | 3.51  (3.22, 3.81) | 3.63  (3.34, 3.92) | 3.38  (2.76, 3.99) |
| 6. I am confident in my ability to recognize artifacts on US images relevant to MSK | 2.42  (2.08, 2.76) | 3.5  (2.41, 4.59) | 2.17  (1.87, 2.48) | 2.48  (2.02, 2.94) | 2.31  (1.77, 2.85) |
| 7. I am confident in my ability to handle the US transducer and obtain US images of the knee (utilizing depth, gain, focus) | 2.60  (2.25, 2.95) | 3.63  (3.00, 4.25) | 2.37  (2.00, 2.74) | 2.59  (2.11, 3.07) | 2.63  (2.08, 3.17) |
| 8. I am confident in my ability to handle the US transducer and obtain US images of the shoulder (utilizing depth, gain, focus) | 2.58  (2.26, 2.90) | 3.5  (2.87, 4.13) | 2.37  (2.04, 2.71) | 2.63  (2.20, 3.06) | 2.5  (1.99, 3.01) |
| 9. I am confident in my understanding of the basic anatomy of the knee | 3.37  (3.04, 3.71) | 3.5  (2.87, 4.13) | 3.34  (2.94, 3.74) | 3.26  (2.80, 3.72) | 3.56  (3.05, 4.08) |
| 10. I am confident in my understanding of the basic anatomy of the shoulder | 3.74  (3.45, 4.04) | 4 | 3.69  (3.32, 4.05) | 3.59  (3.16, 4.02) | 4  (3.66, 4.34) |
| 11. I am confident in my ability to perform physical exam maneuvers for the knee | 2.77  (2.47, 3.07) | 2.63  (2.00, 3.25) | 2.8  (2.45, 3.15) | 2.85  (2.46, 3.24) | 2.63  (2.11, 3.14) |
| 12. I am confident in my ability to perform physical exam maneuvers for the shoulder | 2.84  (2.53, 3.14) | 2.63  (2.19, 3.06) | 2.89  (2.52, 3.26) | 2.81  (2.40, 3.23) | 2.88  (2.36, 3.39) |
| 13. I am confident in my ability to utilize US for supplementing and confirming positive physical exam findings | 2.23  (1.93, 2.54) | 2.5  (1.50, 3.50) | 2.17  (1.84, 2.50) | 2.33  (1.91, 2.76) | 2.06  (1.61, 2.52) |
| 14. US will play a role in the future of anatomy education | 4.23  (4.02, 4.44) | 4.25  (3.86, 4.64) | 4.23  (3.98, 4.48) | 4.37  (4.10, 4.64) | 4  (3.66, 4.34) |
| 15. US is a useful skill for graduates regardless of future specialty | 4.35  (4.08, 4.62) | 4.63  (4.19, 5.06) | 4.29  (3.97, 4.60) | 4.52  (4.22, 4.82) | 4.06  (3.53, 4.59) |
