# Supplementary material for: Unlocking Musculoskeletal Anatomy: Enhancing Second-Year Medical Students’ Knowledge Recall and Self-Efficacy with a Physician-Led Ultrasound Session
Source: Med Sci Educ. 2025 May 20;35(4):2063–74. doi: 10.1007/s40670-025-02414-8 (PMC12532992; doi:10.1007/s40670-025-02414-8)
Supplement: Supplementary file 6 — Supplementary file6 (DOCX 22 KB) [file 40670_2025_2414_MOESM6_ESM.docx]

Article Title - Unlocking Musculoskeletal Anatomy: Enhancing Second-Year Medical Students’ Knowledge Recall and Self-Efficacy with a Physician-Led Ultrasound Session

Journal Name – Medical Science Educator

Author Names – Nathan Cowan, BS^;^ Abdus Sattar, PhD, LLM; Qian Wu, BMS; Allison N. Schroeder, MD

Corresponding Author E-Mail & Affiliation – [aschroe1@alumni.nd.edu](mailto:aschroe1@alumni.nd.edu) ; Department of Physical Medicine & Rehabilitation, MetroHealth Systems, Case Western Reserve University

**Supplemental Material 6**

*Descriptive Statistics of the Post-Session Self-Efficacy Questionnaire for All Participants with Stratification by Ultrasound Elective Membership and Lecture Viewership*

Results are reported as the Mean (95% Confidence Interval)

| **Item** | **Overall** | **US Elective Members** | **US Elective Non-Members** | **Lecture Viewers** | **Lecture Non-Viewers** |
| --- | --- | --- | --- | --- | --- |
| 1. As of today, I feel confident in my ability to differentiate various normal tissues and anatomic landmarks of the knee based on palpation/visual inspection | 3.86  (3.63, 4.09) | 4.25  (3.66, 4.84) | 3.77  (3.52, 4.02) | 3.89  (3.64, 4.14) | 3.81  (3.33, 4.30) |
| 2. As of today, I feel confident in my ability to differentiate various normal tissues and anatomic landmarks of the shoulder based on palpation/visual inspection | 3.86  (3.61, 4.11) | 4.25  (3.66, 4.84) | 3.77  (3.49, 4.05) | 3.96  (3.71, 4.22) | 3.69  (3.15, 4.23) |
| 3. As of today, I feel confident in my ability to differentiate various normal tissues and anatomic landmarks of the knee using point of care ultrasound | 3.67  (3.41, 3.94) | 3.75  (3.16, 4.34) | 3.66  (3.35, 3.97) | 3.70  (3.38, 4.03) | 3.63  (3.11, 4.14) |
| 4. As of today, I feel confident in my ability to differentiate various normal tissues and anatomic landmarks of the shoulder using point of care ultrasound | 3.65  (3.39, 3.91) | 3.88  (3.34, 4.41) | 3.6  (3.30, 3.90) | 3.70  (3.44, 3.97) | 3.56  (2.98, 4.15) |
| 5. I understand the basic physics underlying ultrasound as an imaging modality | 3.86  (3.66, 4.06) | 4.25  (3.66, 4.84) | 3.77  (3.57, 3.98) | 3.93  (3.68, 4.17) | 3.75  (3.39, 4.11) |
| 6. I am confident in my ability to recognize artifacts on ultrasound images relevant to MSK | 3.47  (3.17, 3.76) | 4.38  (3.94, 4.81) | 3.26  (2.94, 3.57) | 3.44  (3.04, 3.85) | 3.5  (3.02, 3.98) |
| 7. I am confident in my ability to handle the ultrasound transducer and obtain ultrasound images of the knee (utilizing depth, gain, focus) | 3.84  (3.56, 4.11) | 4.38  (3.75, 5.00) | 3.71  (3.41, 4.02) | 3.85  (3.51, 4.19) | 3.81  (3.29, 4.34) |
| 8. I am confident in my ability to handle the ultrasound transducer and obtain ultrasound images of the shoulder (utilizing depth, gain, focus) | 3.77  (3.49, 4.04) | 4.25  (3.66, 4.84) | 3.66  (3.35, 3.97) | 3.85  (3.51, 4.19) | 3.63  (3.11, 4.14) |
| 9. I am confident in my understanding of the basic anatomy of the knee | 4.02  (3.78, 4.27) | 4.25  (3.86, 4.64) | 3.97  (3.68, 4.27) | 4  (3.69, 4.31) | 4.06  (3.61, 4.52) |
| 10. I am confident in my understanding of the basic anatomy of the shoulder | 4.09  (3.88, 4.30) | 4.38  (3.94, 4.81) | 4.03  (3.79, 4.27) | 4  (3.71, 4.29) | 4.25  (3.94, 4.56) |
| 11. I am confident in my ability to perform physical exam maneuvers for the knee | 3.30  (3.01, 3.59) | 3.75  (3.16, 4.34) | 3.2  (2.87, 3.53) | 3.22  (2.85, 3.59) | 3.44  (2.92, 3.95) |
| 12. I am confident in my ability to perform physical exam maneuvers for the shoulder | 3.56  (3.28, 3.84) | 3.75  (3.16, 4.34) | 3.51  (3.19, 3.84) | 3.37  (3.00, 3.74) | 3.88  (3.45, 4.30) |
| 13. I am confident in my ability to utilize ultrasound for supplementing and confirming positive physical exam findings | 3.49  (3.17, 3.81) | 4  (3.23, 4.77) | 3.37  (3.02, 3.73) | 3.59  (3.18, 4.01) | 3.31  (2.77, 3.85) |
| 14. Ultrasound will play a role in the future of anatomy education | 4.44  (4.26, 4.62) | 4.5  (4.05, 4.95) | 4.43  (4.22, 4.64) | 4.52  (4.29, 4.75) | 4.31  (3.99, 4.63) |
| 15. Ultrasound is a useful skill for graduates regardless of future specialty | 4.40  (4.19, 4.60) | 4.63  (4.19, 5.06) | 4.34  (4.11, 4.58) | 4.56  (4.36, 4.76) | 4.13  (3.70, 4.55) |
| 16. The ultrasound workshop helped improve my comprehension of the anatomy of the knee | 4.14  (3.94, 4.34) | 4  (3.37, 4.63) | 4.17  (3.96, 4.38) | 4.19  (3.96, 4.41) | 4.06  (3.65, 4.47) |
| 17. The ultrasound workshop helped improve my comprehension of the anatomy of the shoulder | 4.09  (3.87, 4.31) | 4.13  (3.59, 4.66) | 4.09  (3.83, 4.34) | 4.15  (3.91, 4.39) | 4  (3.52, 4.48) |
| 18. The ultrasound workshop helped improve my physical examination skills for the knee | 3.56  (3.24, 3.88) | 3.38  (2.38, 4.37) | 3.6  (3.25, 3.95) | 3.56  (3.13, 3.99) | 3.56  (3.05, 4.08) |
| 19. The ultrasound workshop helped improve my physical examination skills for the shoulder | 3.56  (3.25, 3.87) | 3.25  (2.38, 4.12) | 3.63  (3.28, 3.97) | 3.52  (3.10, 3.93) | 3.63  (3.11, 4.14) |
| 20. The ultrasound workshop improved my ability to orient myself to a sonographic image of the knee | 4.21  (4.01, 4.41) | 4.13  (3.59, 4.66) | 4.23  (4.01, 4.45) | 4.30  (4.06, 4.54) | 4.06  (3.70, 4.42) |
| 21. The ultrasound workshop improved my ability to orient myself to a sonographic image of the shoulder | 4.19  (3.99, 4.38) | 4.13  (3.59, 4.66) | 4.2  (3.98, 4.42) | 4.26  (4.02, 4.49) | 4.06  (3.70, 4.42) |
| 22. The amount of instruction provided prior to and during the MSK ultrasound session was adequate | 3.91  (3.62, 4.19) | 4.13  (3.83, 4.42) | 3.86  (3.51, 4.20) | 3.81  (3.45, 4.18) | 4.06  (3.57, 4.56) |
| 23. This session's content was relevant to my learning and added value to my Block 5 GARLA | 4.40  (4.23, 4.56) | 4.38  (3.94, 4.81) | 4.4  (4.21, 4.59) | 4.48  (4.28, 4.68) | 4.25  (3.94, 4.56) |
