# Supplementary material for: Unlocking Musculoskeletal Anatomy: Enhancing Second-Year Medical Students’ Knowledge Recall and Self-Efficacy with a Physician-Led Ultrasound Session
Source: Med Sci Educ. 2025 May 20;35(4):2063–74. doi: 10.1007/s40670-025-02414-8 (PMC12532992; doi:10.1007/s40670-025-02414-8)

Article Title - Unlocking Musculoskeletal Anatomy: Enhancing Second-Year Medical Students’ Knowledge Recall and Self-Efficacy with a Physician-Led Ultrasound Session

Journal Name – Medical Science Educator

Author Names – Nathan Cowan, BS^;^ Abdus Sattar, PhD, LLM; Qian Wu, BMS; Allison N. Schroeder, MD

Corresponding Author E-Mail & Affiliation – [aschroe1@alumni.nd.edu](mailto:aschroe1@alumni.nd.edu) ; Department of Physical Medicine & Rehabilitation, MetroHealth Systems, Case Western Reserve University

**Supplemental Material 7**

*Content Assessment Score Distributions for Control and Experimental Groups Stratified by Lecture Viewership*


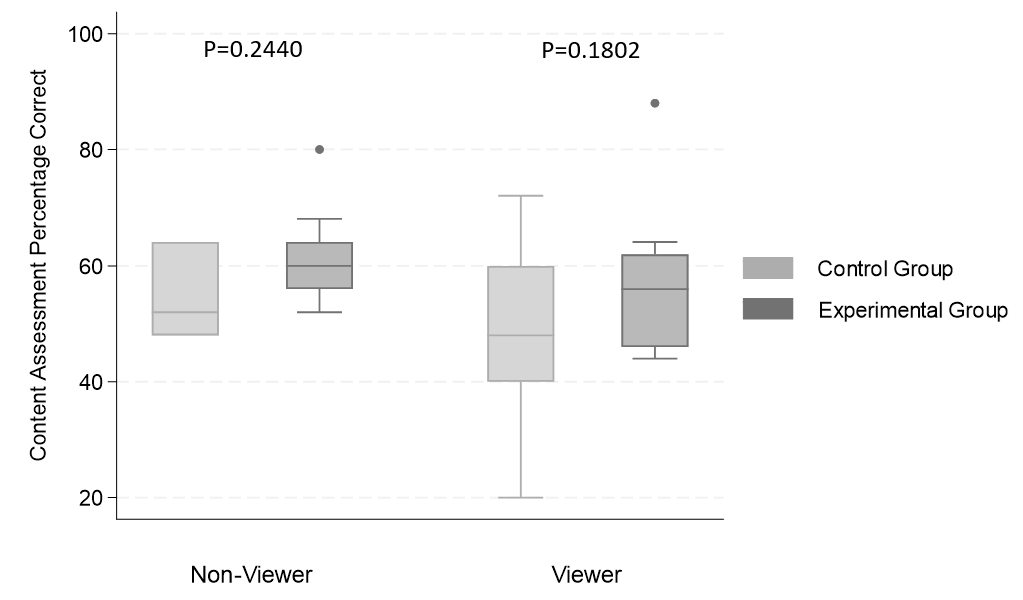

Supplement: Supplementary file 7 — Supplementary file7 (DOCX 48 KB) [file 40670_2025_2414_MOESM7_ESM.docx]
